# CLEAR: Multimodal Human Activity Recognition via Contrastive Learning Based Feature Extraction Refinement

**DOI:** 10.3390/s25030896

**Published:** 2025-02-01

**Authors:** Mingming Cao, Jie Wan, Xiang Gu

**Affiliations:** 1 School of Information Science and Technology, Nantong University, Nantong 226001, China; 2230310047@stmail.ntu.edu.cn; 2 School of Artificial Intelligence and Computer Science, Nantong University, Nantong 226001, China; gu.x@ntu.edu.cn

**Keywords:** HAR, contrastive learning, multimodal, domain generalization

## Abstract

Human activity recognition (HAR) has become a crucial research area for many applications, such as Healthcare, surveillance, etc. With the development of artificial intelligence (AI) and Internet of Things (IoT), sensor-based HAR has gained increasing attention and presents great advantages to existing work. Relying solely on existing labeled data may not adequately address the challenge of ensuring the model’s generalization ability to new data. The ’CLEAR’ method is designed to improve the accuracy of multimodal human activity recognition. This approach employs data augmentation, multimodal feature fusion, and contrastive learning techniques. These strategies are utilized to refine and extract highly discriminative features from various data sources, thereby significantly enhancing the model’s capacity to identify and classify diverse human activities accurately. CLEAR achieves high generalization performance on unknown datasets using only training data. Furthermore, CLEAR can be directly applied to various target domains without retraining or fine-tuning. Specifically, CLEAR consists of two parts. First, it employs data augmentation techniques in both the time and frequency domains to enrich the training data. Second, it optimizes feature extraction using attention-based multimodal fusion techniques and employs supervised contrastive learning to improve feature discriminability. We achieved accuracy rates of 81.09%, 90.45%, and 82.75% on three public datasets USC-HAD, DSADS, and PAMAP2, respectively. Additionally, when the training data are reduced from 100% to 20%, the model’s accuracy on the three datasets decreases by only about 5%, demonstrating that our model possesses strong generalization capabilities. Additionally, when the training data are reduced from 100% to 20%, the model’s accuracy on the three datasets decreases by only about 5%, demonstrating that our model possesses strong generalization capabilities.

## 1. Introduction

HAR has become one of the most crucial technologies in many real-world applications, such as healthcare, smart homes and sports monitoring [1]. HAR aims to detect human movement features through various data signals, and analyze and identify the current activity status. HAR can be briefly categorized as vision-based HAR and sensor-based HAR [2,3]. To identify the activity of daily living, vision-based human activity recognition is often criticized for its privacy issues and environmental limitations. On the other hand, due to the rapid progress of mobile sensing and ubiquitous computing, sensor-based HAR [4] can reduce reliance on environmental factors, enhance privacy protection, and broaden its application domains. We focus on sensor-based HAR in this work.

Currently, sensor-based HAR primarily relies on machine learning or deep learning techniques [5,6] for model construction. Traditional machine learning methods such as k-nearest neighbors and Support Vector Machines (SVM) utilize handcrafted features to recognize activities. However, due to limitations in expert experience and domain knowledge, only shallow feature representations can be obtained, resulting in decreased classification accuracy. On the other hand, deep neural networks including Convolutional Neural Networks (CNNs), Recurrent Neural Networks (RNNs), and Long Short-Term Memory (LSTM) networks exhibit significant capability in extracting meaningful feature representations [7,8]. CNNs can effectively extract spatial features with their multi-scale representation capability; RNNs can utilize memory functions to capture contextual information in time-series data and LSTMs model long-term temporal dependencies through gate mechanisms. Typically, a combination of these models is employed in practice to improve the capture capability of effective features from time-series data.

**Figure 1:**
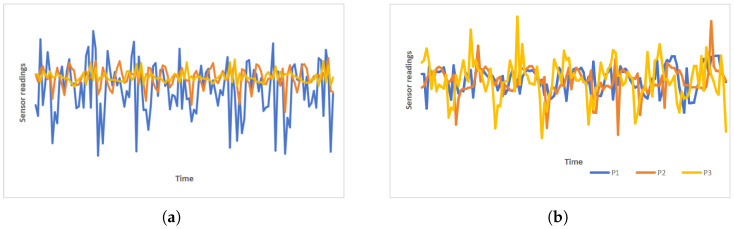
(**a**) A person’s activity patterns vary throughout different time periods. (**b**) Different individuals exhibit varying ways of engaging in the same activity.

**Figure 2:**
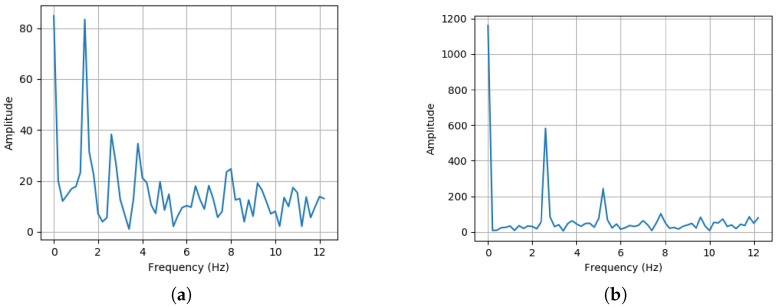
Different individuals exhibit varying ways of engaging in the same activity.

Despite great progress, current HAR still faces two critical challenges that prevent us from building a generalizable model to perform well on unseen data. First, compared to the image domain, annotating time-series data often requires prior knowledge of each time segment [9], significantly increasing the cost of acquiring training data. Second, as shown in Figure 1 and Figure 2, datasets from different individuals may exhibit different distributions in both the time domain and the frequency domain, which may further worsen the challenge of generalization. Figure 1 represents the accelerometer sensor signal during the activity of running from the DSADS [10] dataset, and Figure 2 represents the spectral representation of the corresponding sensor signal. Specifically, the first subgraph in Figure 1 illustrates how the sensor data distribution changes over time for the same individual performing the same activity, while the second subgraph in Figure 1 highlights the differences in sensor data distribution among different individuals engaged in the same activity. When training data are insufficient, the model’s generalization ability may be limited [11,12]. To alleviate the negative impact of these factors on model generalization, research on DA and Domain Generalization(DG) in HAR has acquired significant attention in recent years [13]. DA aims to adapt a model from its original domain to another domain. Models are trained using data from the source domain and then fine-tuned on the target domain to better adapt to the data distribution of the target domain. Techniques commonly used in DA include feature alignment and adaptive training methods. However, DA typically requires access to target data during the training phase, which is often impractical in real-world applications, posing a major challenge to improving model generalization capability. On the other hand, DG focuses on improving the model’s ability to generalize to different but related domains, without relying on domain-specific knowledge. The objective of DG is to train models to exhibit robustness to variations in data distribution encountered during the training process, thereby empowering them to generalize effectively to unobserved domains.

In this study, our objective is to improve the model’s generalization ability by broadening the distribution of training domain data and improving feature discriminability. Initially, we perform data augmentation in both the time domain and frequency domain to mitigate distribution shift challenges between the training domain and the target domain induced by inadequate training data. Following that, capitalizing on the intrinsic multimodal characteristics of data gathered from various sensors, we utilize CNNs to extract features independently from each modality, followed by the fusion of these features to obtain more effective representations. Furthermore, to improve feature discriminability, we introduce contrastive learning methodologies to further refine the model’s generalization capabilities. This sequential approach aims to construct a comprehensive and effective model framework to mitigate the impact of distribution shift issues on HAR.

We present several novel contributions in this work:

• Extensive augmented data are obtained from multiple perspectives by fine-tuning the amplitude, frequency, and phase of the data in the frequency domain. Combined with time-domain augmentation techniques, we have significantly enriched the training dataset. This strategy notably enhances the model’s ability to generalize across various activity patterns.
• We effectively leverage the multimodal characteristics of sensor data by implementing convolutional subnetworks and attention mechanisms for localized feature extraction and adaptive feature fusion. It improves feature representativeness and performance in downstream classification tasks.
• We introduce an enhanced contrastive learning method that improves feature discriminability by strategically selecting contrastive samples near the classification boundary. This approach reduces computational costs while facilitating the learning of discriminative inter-class features.
• We conducted extensive experiments on three publicly available datasets. The experimental results clearly demonstrate that CLEAR exhibits superior generalization performance in addressing distribution shift issues, resulting in a significant improvement in accuracy.

This paper is organized as follows: Section 2 presents a literature review of sensor-based HAR. In Section 3, a comprehensive description of CLEAR is demonstrated. Section 4 shows how we conducted the experiment and evaluation with three benchmark datasets. Finally, Section 5 concludes the paper and presents insights into potential avenues for future research.

## 2. Related Work

### 2.1. Human Activity Recognition

HAR is a field of study within computer science and AI that focuses on the development of algorithms and techniques to identify, classify, and understand human activities based on data collected from various sensors. The goal of HAR is to automatically recognize and interpret human actions using data from sensors such as accelerometers, gyroscopes, magnetometers, and sometimes even cameras. Existing HAR methods mainly fall into two categories: vision-based HAR and sensor-based HAR. Furthermore, due to the rapid development of wearable technology and the IoT, sensor-based HAR has become the predominant research area. This approach provides advantages including reduced dependence on environmental light, enhanced privacy protection capabilities and decreased computational overheads. This work is focused on sensor-based HAR.

Numerous machine learning-based methodologies have been proposed to tackle the HAR problem. Traditional approaches typically rely on statistical knowledge [14,15] for feature construction. In recent years, rapidly evolving deep learning techniques [16,17] have provided more convenient and efficient methods for feature extraction, effectively improving model recognition accuracy. Additionally, some scholars [18] have achieved encouraging results by integrating statistical methods with deep learning techniques. With the development of image classification techniques, some studies [19,20] transform sequential data into image data for feature extraction. However, the majority of the existing works assume that the training domain dataset and the target domain dataset have identical distributions. In the real world, however, the scarcity of training data leads to a significant domain distribution shift between training and testing sets [21], which severely restricts the generalization ability [22,23] of HAR models. Several semi-supervised learning-based strategies have been proposed, facilitating more effective utilization of unlabeled data for feature extraction [24,25].

### 2.2. Multimodal Human Activity Recognition

Multimodal Human Activity Recognition (MMHAR) builds upon the concept of HAR by incorporating data from multiple sources or modalities to improve the accuracy and robustness of activity recognition systems. Instead of relying solely on data from a single type of sensor, such as accelerometers or gyroscopes, MMHAR integrates information from various sensors as well as other data sources like audio, video, and environmental sensors. The process of MMHAR often involves a few steps, such as data fusion, feature fusion, model fusion and Evaluation. Data fusion integrates multimodal data from different sensors or data sources to create a comprehensive dataset; feature fusion combines features extracted from multiple modalities, such as video, audio, inertial sensors, etc., to learn effective features for downstream tasks from multiple perspectives; model fusion combines multiple models or algorithms to improve performance; finally, the model’s performance is evaluated using independent testing datasets, assessing metrics such as accuracy, recall, and others. Multimodal fusion techniques have wide applications in image processing, natural language processing, and HAR.

In recent years, there have been several multimodal studies on sensor-based HAR. The principle of “sufficient gathering and judicious selection” proposed by Chen [26] aims to adopt the multimodal nature of wearable sensors for extracting more effective and comprehensive feature representations. Initially, sensor data undergoes a transformation into a two-dimensional matrix. Subsequently, an interpretable parallel recurrent model is utilized for activity recognition, comprising an activity frame-based LSTM and an attention-based LSTM. Activity frames are formed by stacking the raw signals row-wise and then using permutations to place the data from each sensor adjacent to data from other sensors. In this model, the activity frame captures modal features and relationships between feature pairs. The model utilizes a Glimpse Network which can gradually process data across multiple time steps, thereby mitigating information loss. Utilizing attention mechanisms, the model extracts and precisely selects the most salient regions from multimodal sensor data, thus maximizing the utilization of effective features while minimizing computational costs. The model’s performance is evaluated against four state-of-the-art multimodal feature-based methods using the PAMAP2 and MHEALTH datasets. The results demonstrate that the model achieves competitive performance on two benchmark datasets and works well in real-life scenarios. However, the sensitivity of model hyperparameter selection, such as the size of the glimpse window, the number of Monte Carlo samples, the quantity of glimpses, and the high reliance on training data, constrains the model’s generalization ability to unknown data. COCOA [27] introduces a novel approach aimed at learning effective feature representations from data across multiple sensor modalities. Compared to traditional contrastive learning methods [28], it offers several advantages and distinctive features. Firstly, it adopts a broader interpretation of the term “modality”, encompassing various types of data, sensors, and input channels. Secondly, it is the pioneering method to apply contrastive learning to multiple modalities simultaneously. By leveraging inter-modal mutual information as supervisory signals, it effectively exploits the associations between diverse data sources. The method comprises two primary components; it utilizes modality-specific encoders to transform heterogeneous input data into a shared latent space. Then, in the training phase, modality fusion is achieved by employing a single dense projection head for each modality along with a shared dense layer. Extensive experimentation evaluated COCOA against supervised approaches and five SSL objective functions, confirming its superiority in acquiring valuable representations for downstream classification tasks. However, COCOA utilizes all data to construct positive and negative sample pairs, which not only increases computational burdens but also lacks robustness towards noise, outliers, or incomplete data within the dataset.

Jain et al. proposed a collaborative self-supervised learning approach [29], extending traditional self-supervised learning algorithms to multi-device environments through three technical innovations: selecting positive and negative devices to enable contrastive learning, sampling positive and negative samples in multi-device environments, and a loss function for multi-view contrastive loss.

### 2.3. Data Augmentation Improves Generalization by Enriching Data Diversity

Data augmentation is a technique used in machine learning and data science to artificially increase the size of a training dataset by creating modified versions of the existing data. The goal of data augmentation is to improve the performance and generalization of machine learning models, especially when the original dataset is limited in size. The shortage of training data [30,31] is a critical factor impacting the performance of deep learning models [32]. Data augmentation [33] techniques play a vital role across various domains, including image processing and HAR. Additionally, data augmentation diversifies the training dataset, reducing the model’s dependence on specific instances and mitigating the risk of overfitting. Carefully selecting and applying data augmentation methods [34] can significantly optimize the performance and robustness of the model, enabling it to better handle complex real-world data.

CutMix [35] offers an effective augmentation strategy by cutting and pasting image patches and mixing labels based on patch areas. This approach enhances pixel utilization while maintaining the regularization benefits of region dropout. This method reduces the risk of overfitting, directs the model’s focus to less noticeable object parts, and enhances generalization and localization in CNNs. It has notably improved classification accuracy on ImageNet and CIFAR datasets. It also enhances classifier robustness and uncertainty management. However, CutMix may increase computational demands and introduce stability issues, requiring careful tuning of parameters and loss functions for broader applicability. Conversely, FIXED [36] adopts Domain Adversarial Neural Networks (DANN) [37] to learn domain-invariant features through adversarial training. It improves on Mixup [33] by applying it to these features and incorporating a large margin loss to mitigate the impact of synthetic noise. This method enhances model robustness and generalization, demonstrating flexibility across various classification tasks and ease of integration into existing domain generalization (DG) frameworks. However, the effectiveness of FIXED depends on the suitability of the augmentation approach, as poor strategy selection may compromise generalization performance.

Bakhshayesh et al. [38] evaluated and compared six data augmentation methods for classification using ConvLSTM networks, namely autoencoders, time warping, amplitude warping, scaling, jittering, and linear combinations. It demonstrates the positive influence of data augmentation on the recognition accuracy of HAR models. Mixup [39] introduces a novel data augmentation technique by performing linear interpolation on input data to generate new training samples, thereby augmenting the training dataset. Furthermore, it demonstrates the effectiveness of mixup in reducing model generalization error and improving model robustness, surpassing traditional empirical risk minimization methods.

Frequency domain information, like time domain information, contains many effective representation features. Time domain features capture overall trends and fluctuations, while frequency domain features reveal data components across different frequencies. However, most studies focus solely on time domain features, with limited research on frequency domain feature extraction or their combination. Integrating augmentations from both domains can significantly enhance data diversity and provide a more comprehensive understanding of its underlying characteristics.

### 2.4. Contrastive Learning Increases Feature Distinguishability to Optimize Model Performance

Contrastive learning is a widely used unsupervised learning technique in deep learning that compares the similarity and dissimilarity between different samples for feature representation [40]. This approach typically does not only depend on labeled data [41]; instead, it leverages relationships between samples to facilitate the learning of more meaningful feature representations. Contrastive learning comprises three key elements: anchor, positive samples, and negative samples. The anchor, derived from the original data, acts as the reference point for constructing and selecting positive and negative samples. Positive samples consist of one or more instances generated [42] using data augmentation techniques, designed to be similar to the anchor. These augmented samples are assumed to possess similar feature representations to the original sample since they stem from the same data point. On the other hand, negative samples are typically chosen from data points distinct from the anchor. The selection of positive and negative samples and the design of the loss function are crucial factors influencing the effectiveness of contrastive learning.

TS-TCC [43] utilizes contrastive learning for extracting robust and discriminative representations from time series data. It designs specialized weak jitter-and-scale and strong permutation-and-jitter augmentation techniques for time series data, thereby transforming raw time series data into two views. Afterward, it introduces a temporal contrastive module and a contextual contrastive module. The temporal contrastive module leverages a cross-view prediction task to learn robust representations by incorporating augmented past latent features to predict future features, thereby mitigating the disturbances introduced by varying time steps and augmentation. Meanwhile, the contextual contrastive module further utilizes robust representations to maximize the similarity between different contexts of identical samples while minimizing the similarity between contexts of distinct samples. TS-TCC possesses unique advantages in constructing diverse views and addresses the complexity and variability issues of time series data. The experiments demonstrate that TS-TCC performs well on classification tasks. However, TS-TCC may depend on specific types of data augmentation, which limits its generalization capability on unknown datasets. TS-CoT [44] presents a novel approach in time series representation learning, focusing on the robust handling of noisy and corrupted data within a multi-view setting. It employs single-view encoders to generate latent representations of data, utilizes data augmentation to create positive pairs, and calculates contrastive loss to optimize the encoder. It describes the semantic structure of different views using prototypes, which represent cluster centers in the feature space. TS-CoT adopts a prototype-based contrastive co-training loss and a moving average prototype update strategy to stabilize the learning process. Effectively integrating complementary information from different views, it enhances the quality of time series data representation through multi-view learning, particularly in the presence of noise. However, the initialization of prototypes in the TS-CoT model impacts both the subsequent training process and the overall quality of feature representation, making it challenging to directly apply to new datasets.

Recently, several studies have integrated contrastive learning into supervised learning. With known data labels, the formation of positive and negative sample pairs becomes more straightforward: samples sharing the same label as the anchor are considered positive, while those with distinct labels are considered negative. The primary objective of contrastive learning is to train an encoder that transforms input data into a feature space where similar samples are drawn closer together and dissimilar ones are pushed further apart. Khosla et al. introduced a loss function named SupCon [45], which improves traditional contrastive loss functions by allowing each anchor to have multiple positive samples, thereby satisfying the demands of supervised learning. Lan et al. performed a thorough analysis of how bad-positive pairs affect feature quality in time series contrastive learning [46], considering both theoretical and empirical viewpoints. They introduced the Dynamic Bad Pair Mining algorithm to mitigate this problem. The main challenge in supervised contrastive learning lies in selecting positive and negative sample pairs [47]. Having an excessive number of positive and negative samples can result in issues such as high computational complexity and convergence difficulties. Conversely, having too few positive and negative samples may decrease the model’s performance and generalization ability.

## 3. Methodology

Wearable sensor-based HAR aims at identifying specific movements and behaviors utilizing sensor signals. It achieves automatic recognition of daily activities, exercise postures, etc., through the collection and analysis of sensor data, providing crucial technical support for fields such as health monitoring, sports training, behavior analysis, and more. However, most current models fail to account for the unpredictable diversity present in real-world data, which decreases the ability to generalize when trained and tested on the same dataset. CLEAR effectively alleviates distributional shifts, thereby improving the model’s generalization capability to unknown datasets.

### 3.1. Problem Definitions

Domain can be defined as a joint probability distribution $P_{X,Y}$ over X×Y, where *X* and *Y* represent the instance space and label space, respectively. During the training phase, a labeled dataset $D_{labled}$ is utilized as the training set. The objective of DG is to obtain suitable model parameters through the training set, enabling the model to perform well on an unknown dataset $D_{unlabled}$. However, due to the intrinsic characteristics of time series data, annotating samples is time and cost-consuming. Moreover, for deep learning-based models, the amount of training data often significantly impacts performance. This ultimately results in the model’s generalization ability being less than ideal when applied to new data.

Previous methods for DG typically assumed that the target domain and training domain have the same feature set and label spaces, where $X^{source}=X^{target}$, $Y^{source}=Y^{target}$, $P^{s}\left(x_{i},y_{i}\right)=P^{t}\left(x_{i},y_{i}\right)$. However, in practice, due to differences in underlying factors such as gender, age, and habits, among subjects there often exists the domain distribution shift (DDS) between different datasets, i.e., $X^{source}=X^{target}$, $Y^{source}=Y^{target}$, $P^{s}\left(x_{i},y_{i}\right)\neq P^{t}\left(x_{i},y_{i}\right)$. This inevitably leads to the necessity when designing a model to consider not only its accuracy on given labeled data but also how to utilize existing labeled data to ensure its accuracy when applied to new data, i.e., the generalization capability.

### 3.2. The Overall Structure of CLEAR

**Figure 3:**
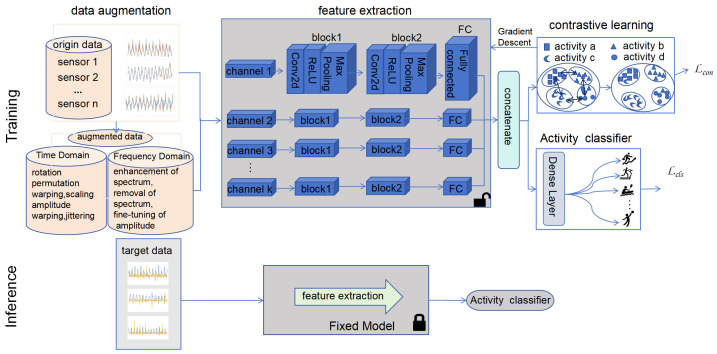
The Overall Framework of CLEAR. The upper half of the figure represents the training section, while the lower half represents the testing section.

As depicted in Figure 3, the CLEAR framework consists of two phases: training and testing, where each module works in close collaboration to achieve efficient feature learning and classification. During the training phase, the raw data are first preprocessed using data augmentation techniques in both the time and frequency domains (e.g., rotation, permutation, and adjustments to amplitude and frequency) to enhance data diversity and robustness, thereby laying the foundation for subsequent feature extraction. Subsequently, a multimodal fusion approach is employed to integrate data from different sensors, extracting more discriminative feature representations through feature-level fusion, which improves the model’s adaptability to complex scenarios. Building on this, a contrastive learning mechanism is introduced to further enhance feature discriminability by optimizing the aggregation of similar samples and the separation of dissimilar ones, significantly improving the accuracy and robustness of classification tasks. Finally, a single-layer linear classifier is used to categorize the learned features, enabling the precise recognition of different activity classes. During the inference phase, the model loads the optimized parameters obtained during training to evaluate the test set data, validating its generalization ability and classification performance in real-world scenarios. This modular design not only ensures efficient collaboration among components but also provides flexibility for model expansion and optimization.

### 3.3. Data Augmentation Enriches the Data Distribution

In the domain of visual perception, data augmentation techniques have achieved a notable degree of maturity. However, compared to image data, time series features not only contain spatial information [48] but also crucially depend on their temporal dependencies [49,50,51]. Therefore, there is a need to explore data augmentation methods suitable specifically for time series data. The temporal domain of a sequence illustrates how sensor readings change over time, while the frequency domain indicates the magnitude of signals within each frequency component of the entire spectrum. Considering the frequency domain can offer an alternative perspective for understanding time series information, thus enabling a more comprehensive utilization of labeled sample information.

Regarding time domain data augmentation techniques, we utilize rotation, permutation, time warping, scaling, jittering, and random sampling as shown in Equation (1). Where $x^{\prime }$ denotes the resultant vector obtained after a series of transformations; $x_{f(k)}$ signifies the k-th element in the result of applying the function *f* to vector *x*; $x_{\pi (2)}$ represents the k-th element selected from vector *x* according to the permutation function π; *R* is a matrix utilized for linear transformations of vector *x*; $c_{n}$ is a constant employed for scaling the elements of vector *x*; $x_{i_{m}}$ denotes the m-th element selected from vector *x*, where $i_{m}$ denotes the index of the selected element.

(1) $$\begin{array}{cc} {x^{\prime }}_{1} & =Rx\;{x^{\prime }}_{2}=\left(x_{\pi (1)},x_{\pi (2)},\ldots ,x_{\pi (n)}\right)\\ {x^{\prime }}_{3} & =(x_{f(1)},x_{f(2)},\ldots ,x_{f(n)})\\ {x^{\prime }}_{4} & =(c_{1}x_{1},c_{2}x_{2},\ldots ,c_{n}x_{n})\\ {x^{\prime }}_{5} & =(x_{1}+\epsilon_{1},x_{2}+\epsilon_{2},\ldots ,x_{n}+\epsilon_{n})\\ {x^{\prime }}_{6} & =(x_{i_{1}},x_{i_{2}},\ldots ,x_{i_{m}}) \end{array}$$

In the frequency domain, the significance of frequency is in revealing the periodicity of activities. The spectrum displays the intensity or energy distribution of different frequency components. Phase informs about periodic pattern positions on the time axis and time delay relationships among different frequency components, describing temporal relationships between activities. Amplitude represents the magnitude of periodic patterns in the time series data, i.e., the intensity of activities. The integrated utilization of this information contributes to a more comprehensive understanding of the characteristics of human activities and fits the phenomenon of distribution shift. Therefore, we utilize frequency domain augmentation to improve data diversity.

Firstly, utilize the Fourier transform [52] to convert the signal from the time domain to the frequency domain. According to DIFEX [53], the Fourier transformation F(x) for single-channel two-dimensional data *x* is formulated as Equation (2).

(2) $$F\left(x\right)\left(u,v\right)=\sum_{h=0}^{H-1}\sum_{w=0}^{W-1}x\left(h,w\right)e^{-j2\pi \left(\frac{hu}{H}+\frac{wv}{W}\right)}$$

where *u* and *v* are indices. *H* and *W* are the height and the width, respectively. We utilize the phase offset technique as shown in Equation (3) to adjust the phase of the spectrum. The original data are segmented into samples at fixed time intervals. Phase shifting appropriately modifies the start and end times of each sample, facilitating a deeper understanding of each activity stage.

(3) $$P_{\mathrm{offset}}\left(u,v\right)=\arctan\left(\frac{R(x)(u,v)}{I(x)(u,v)}\right)+\phi_{\mathrm{offset}}$$

where R(x) and I(x) represent the real and imaginary parts of F(x), respectively; $\phi_{\mathrm{offset}}$ is the phase shift. Each frequency component in the spectrum represents a basic function with corresponding frequency and amplitude (e.g., cosine functions in Fourier transform). We employ random addition or deletion as shown in Equation (4) for data augmentation.

(4) $$F^{\prime }\left(u,v\right)=F\left(u,v\right)\pm \frac{1}{HW}x\left(h^{\prime },w^{\prime }\right)e^{-j2\pi \left(\frac{Hu^{\prime }}{H}h^{\prime }+\frac{Wv^{\prime }}{W}w^{\prime }\right)}$$

In the frequency domain, frequency signifies the periodicity, while amplitude denotes the intensity of activities. We increase the data distribution by augmenting and diminishing these parameters, as illustrated in Equation (5).

(5) $$\begin{array}{cc} F_{\mathrm{EF}}\left(u,v\right) & =\sum_{h=0}^{H-1}\sum_{w=0}^{W-1}x\left(h,w\right)e^{-j2\pi (\alpha \frac{hu}{H}+\beta \frac{wv}{W})}\\ F_{\mathrm{EA}}\left(u,v\right) & =\sum_{h=0}^{H-1}\sum_{w=0}^{W-1}\left(\gamma x\left(h,w\right)\right)e^{-j2\pi (\frac{hu}{H}+\frac{wv}{W})} \end{array}$$

where the parameters α, β, and γ are used to fine-tune the magnitude and frequency. Considering that small disturbances in the frequency domain may have significant impacts on the temporal patterns in the time domain, we introduce a parameter μ to control the frequency perturbation. This parameter indicates the number of manipulated frequency components, ensuring that the time series remains similar to the original samples in the time domain after perturbation operations.

### 3.4. Multimodal Feature Fusion

Effective feature extraction is crucial after obtaining appropriate labeled data. Existing models often treat data from multiple wearable sensors as a whole for feature extraction, but overlook local features and correlations. The design of feature extraction modules should consider the high dimensionality and multimodal nature of the data, which is essential for effectively extracting activity features and supporting subsequent classification tasks. Human activities involve various body parts, each with differing importance, resulting in the natural multimodal characteristics of sensor data. CLEAR leverages this characteristic during the feature extraction phase to acquire more comprehensive and effective feature information.

To effectively leverage the multimodal nature of sensor data, we introduce an attention mechanism aimed at extracting features that are more meaningful for classification tasks. Initially, we extract features from each modality individually. Previous studies have shown that CNNs offer significant advantages in processing sensor data. Therefore, we employ a convolutional subnetwork to extract features from each sensor. This subnetwork comprises two stacked convolutional layers and pooling layers, followed by a fully connected layer. Additionally, we apply the Rectified Linear Unit (ReLU) [54] activation function after each CNN layer to enhance the nonlinear mapping for classification tasks. As illustrated in Figure 3, the enhanced data from the *k*th sensor, denoted as $X_{k}$, is fed into the convolutional subnetwork, thus generating a feature vector $F_{k}$ for each sensor.

(6) $$\begin{array}{cc} \zeta_{k} & =\tanh(W_{k}F_{k}+a_{k})\\ \delta_{k} & =\frac{\zeta_{k}w_{k}}{\sum_{k}\alpha_{k}w_{k}}\\ V & =\sum_{k}\delta_{k}F_{k} \end{array}$$

We introduce an attention mechanism to fuse various feature vectors via Equation (6). Firstly, a single-layer perceptron is applied to the feature vector $F_{k}$, resulting in an intermediate vector $\zeta_{k}$. $W_{k}$ represents the weight matrix associated with the feature matrix $F_{k}$, while $a_{k}$ denotes the bias term. Subsequently, we compute the weight vector $delta_{k}$ for the *k*-th sensor relative to the overall features. Finally, based on these weights, the features from each modality are fused to obtain the final feature vector *V*. CLEAR fully exploits the different characteristics of each modality and their interrelationships by extracting features from subsequences and employing adaptive feature fusion, rather than directly extracting features from the data. It improves the efficacy of representing features for subsequent tasks.

### 3.5. Contrastive Learning Increases Discrimination

When the distributions of various activities are expanded through data augmentation techniques, the feature vectors representing each activity in the feature space become denser. This increased density poses challenges for training classification models. Therefore, improving the discriminative capacity of activities within the feature space becomes necessary to maintain the model’s generalization ability. To address this issue, we propose the adoption of contrastive learning.

Contrastive learning was proposed to facilitate three goals: contrasting positive and negative samples, learning similarities, and facilitating self-supervised learning. CLEAR utilizes the task of contrasting positive and negative samples to acquire discriminative feature representations. Initially, we extract feature representations from the samples using the model proposed earlier. Given labeled training data, if other samples within the batch share the same label as the anchor sample, they are considered positive samples; otherwise, they are considered negative samples. Ensuring significant disparities between representations of different classes is crucial for obtaining more discriminative features. Ideally, representations of the same activity should be clustered together, while different activities should be separated from each other. Previous research utilized various distance metrics, such as Euclidean, Manhattan, and Chebyshev distances, as well as cosine similarity (Equation (7)) to measure the similarity between feature vectors. Considering the high-dimensional and large-scale nature of the data, we employ cosine similarity, which is insensitive to vector magnitude, to measure the similarity between feature vectors.

(7) $$\begin{array}{cc} \mathrm{Euclidean}\;\mathrm{Distance} & =\sqrt{\sum_{i=1}^{n}{\left(x_{i}-y_{i}\right)}^{2}}\\ \mathrm{Manhattan}\;\mathrm{Distance} & =\sum_{i=1}^{n}\left|x_{i}-y_{i}\right|\\ \mathrm{Chebyshev}\;\mathrm{Distance} & =\max_{i}\left(\right|x_{i}-y_{i}\left|\right)\\ \mathrm{Cos}\mathrm{ine}\;\mathrm{Similarity} & =\frac{A\cdot B}{∥A∥∥B∥} \end{array}$$

To meet the requirements of the model, we propose $L_{cl}$ via Equation (8) for model training.

(8) $$\begin{array}{cc} \; & L_{cl}\left(v_{i},{\left\{v_{pj}\right\}}_{j=1}^{m},{\left\{v_{nh}\right\}}_{h=1}^{q}\right)=-\log\left(\frac{\sum_{j=1}^{m}\exp\left(\frac{sim(v_{i},v_{pj})}{\tau }\right)}{\sum_{j=1}^{m}\exp\left(\frac{sim(v_{i},v_{pj})}{\tau }\right)+\sum_{h=1}^{q}\exp\left(\frac{sim(v_{i},v_{nh})}{\tau }\right)}\right) \end{array}$$

• $sim(z_{i},z_{j})$ is a function representing the cosine similarity between two samples;
• τ is the temperature parameter;
• $v_{i}$ is the feature representation of the anchor sample;
• ${v_{pj}}_{j=1}^{m}$ is the set containing the feature representations of m positive samples for the anchor sample;
• ${v_{nh}}_{h=1}^{m}$ is the set containing the feature representations of q negative samples for the anchor sample.

The objective of the proposed loss function is to enhance feature distinctiveness by encouraging the convergence of features from the same class while pushing apart those from different classes. The main advantages of $L_{cl}$ over other existing loss functions include the appropriate selection of positive and negative samples to improve the distinctiveness of representation while simultaneously reducing computational burden and accelerating convergence. However, considering the large scale of training data, utilizing all samples within a batch would significantly raise computational complexity and hinder convergence. Hence, it is crucial to select positive and negative samples carefully, especially considering the large scale of training data.

However, in order to improve the recognition accuracy and to further improve the recognition performance, samples that are characterized with low confidence levels must be carefully processed. Such samples often lie in the boundary regions within the feature space, delineating the borders between various class activities. By promoting the convergence of intra-class boundaries and the separation of inter-class feature space boundaries, we can effectively improve feature distinctiveness. To achieve this, positive samples are chosen from those sharing the same label as the anchor but exhibiting lower similarity, while negative samples are selected from those with different labels but showing higher similarity to the anchor. Based on both the set batch size of 128 and adjustments made to parameters during the experimental process, we set the parameters m to 3 and p to 10. During training, the model adjusts its network parameters through backpropagation and gradient descent optimization algorithms to minimize our $L_{cl}$.

### 3.6. Activity Classification

The last step is to classify the activities; this classifier comprises a linear layer that takes the responsibility of mapping features to a one-dimensional vector whose size matches the number of activities. The classification loss $L_{cls}$ is shown in Equation (9).

(9) $$L_{cls}\left(\hat{y},y\right)=-\frac{1}{N}\sum_{i=1}^{N}\sum_{c=1}^{C}y_{ic}\cdot \log\left({\hat{y}}_{ic}\right)$$

where *N* represents the number of samples, *C* represents the number of categories, $\hat{y}$ denotes the output predicted probability distribution, *y* stands for the true category labels, ${\hat{y}}_{ic}$ represents the predicted probability of the *i*-th sample belonging to the *c*-th category, and $y_{ic}$ is an indicator function that equals 1 when the true label of the *i*-th sample belongs to the *c*-th category, and 0 otherwise. The Adam gradient descent optimization algorithm is used to train the model.

## 4. Experiments

### 4.1. Datasets and Experimental Setting

#### 4.1.1. Datasets

To evaluate the performance of CLEAR, experiments were conducted on three benchmark source Wearable Human Activity Recognition datasets:

The DSADS [10] dataset contains sensor sequences gathered from four females and four males aged 20 to 30. It was collected using five Xsens MTx units positioned on the participants’ torso, arms, and legs. The dataset includes 19 activities, spanning from daily routines to personalized exercises, each activity lasting 5 min, sampled at 25 Hz. To facilitate the research for DG, the dataset was divided into four groups, each containing the data sequences from two randomly chosen individuals. Activity signals were segmented into 5-s intervals with an overlap rate of 50% between consecutive windows, resulting in 480 segments per activity where 60 segments per individual.

The USC-HAD dataset [55] comprises seven male and seven female participants, covering 12 distinct behaviors. Each participant performed each behavior five times, with each activity lasting approximately 24 s. Data were collected using a single MotionNode device built into a smartphone pouch, which was worn on the right hip. The device recorded data from its accelerometer and gyroscope at a sampling frequency of 100 Hz. Activity signals were segmented into 5.12 s intervals with an overlap rate of 50% between consecutive windows. To assess the model’s generalization capacity, the participants were categorized into five domains, with each domain containing either three or two participants selected randomly.

The PAMAP2 Physical Activity Monitoring dataset [56] consists of data collected from nine participants, each wearing three inertial measurement units (IMUs) on their hands, chest, and ankles, along with a heart rate monitor. They performed 18 different physical activities, recorded at a sampling frequency of 25 Hz. For the purpose of daily activity recognition, and to simplify the recognition model, only data from IMUs were selected to build and train the model. The dataset from IMUs includes three-axis accelerometers, gyroscopes, and magnetometers. We utilized eight common activities from eight subjects for evaluation: lying, sitting, standing, walking, going up and down the stairs, vacuum cleaning, and ironing. Activity signals were segmented into 5 s intervals with an overlap rate of 50% between consecutive windows. The dataset was partitioned into four domains.

Three datasets’ main statistical information is shown in Table 1. The USC-HAD dataset uses a single accelerometer and gyroscope on the hip, DSADS uses five multi-sensor units (accelerometer, gyroscope, magnetometer) on the torso, arms, and legs, and PAMAP2 uses IMUs with accelerometers, gyroscopes, and magnetometers on the arm, chest, and ankle.

#### 4.1.2. Implementation Details

To prepare the data, we partitioned subjects into separate groups, aiming to streamline the leave-one-out-validation process. This approach constructed an environment conducive to domain-agnostic activity recognition. More precisely, we allocated a specific group of subject data as the focal domain, while the remaining data functioned as the source domain, each delineating a unique objective. For DSADS and PAMAP2, we grouped the eight subjects into four sets, while for datasets involving 14 subjects, we formed five sets. In the latter case, groups 0–3 each comprised three subjects, while the final group included two subjects. Following this, we divided the data within each set into training, validation, and testing subsets in a ratio of 6:2:2. The training set was used for model training, adjusting weights to learn data patterns. The validation set was used for tuning and preventing overfitting by evaluating model performance and selecting the best hyperparameters. The testing set was used for the final evaluation of the model’s generalization ability, ensuring good performance on unseen data. This division helped prevent overfitting, objectively assessing model performance, and optimizing hyperparameters, leading to more reliable and accurate models. We repeated each experiment three times with three different random seeds and took the average as the final result. This approach improved reliability, reduced the impact of randomness, and ensured robust and credible conclusions.

The model’s feature encoder comprised multiple Conv2d layers. For USC-HAD, there were three Conv2d layers with a kernel size of (1,6), whereas for DSADS and PAMAP2, two Conv2d layers with a kernel size of (1,9) were employed. Following each convolutional layer, there was a ReLU activation layer and a MaxPool2d layer. Subsequently, a fully connected layer was utilized for higher-level feature extraction. Finally, behind the feature encoder, multimodal adaptive feature fusion is performed by multiplication with learnable weight parameters. The activity classifier consisted of a fully connected layer with an output dimension equal to the number of activity categories. The coding software we used was ‘PyCharm Community Edition 2021.1.3 x64’, and the Python version was 3.8. The experiments were conducted with a batch size of 128. The number of epochs was 500. The Adam optimizer with a learning rate of $8\times 10^{-4}$ was employed to train the model. The experiments were conducted on an RTX-3070ti GPU.

### 4.2. Experiment Results

Extensive experiments have been conducted with the abovementioned approaches. Building upon the previously mentioned grouping, each group is sequentially designated as the testing domain, while the remaining groups are considered as the source domains for training. To validate the robustness of our model against potential distributional shifts in sensor data collected in real-world scenarios, i.e., its generalization capability, we compare it with recent out-of-distribution (OOD) and semi-supervised learning (SSL) methods on three public datasets. We use accuracy, Precision, Recall and F-measure as the metrics for evaluating the performance of the models, calculated using Equation (10).

(10) $$\begin{array}{cc} Accuracy & =\frac{TP+TN}{TP+FP+FN+TN}\\ Precision & =\frac{TP}{TP+FP}\\ Recall & =\frac{TP}{TP+FN}\\ F-measure & =\frac{2\times Precision\times Recall}{Precision+Recall} \end{array}$$

where TP denotes true positives, TN denotes true negatives, FP denotes false positives, and FN denotes false negatives. These statistical metrics indicate the accuracy and effectiveness of CLEAR.

**Figure 4:**
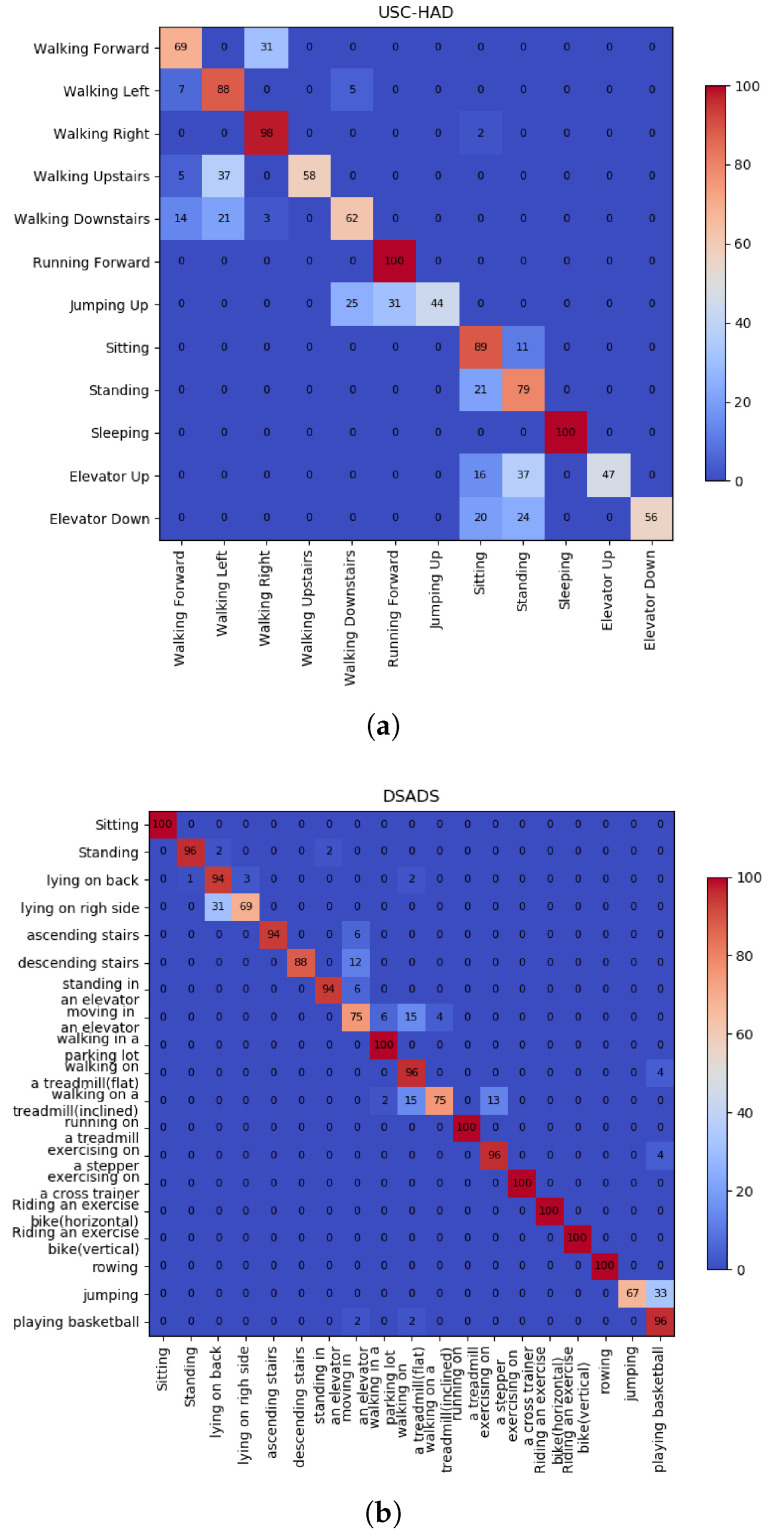

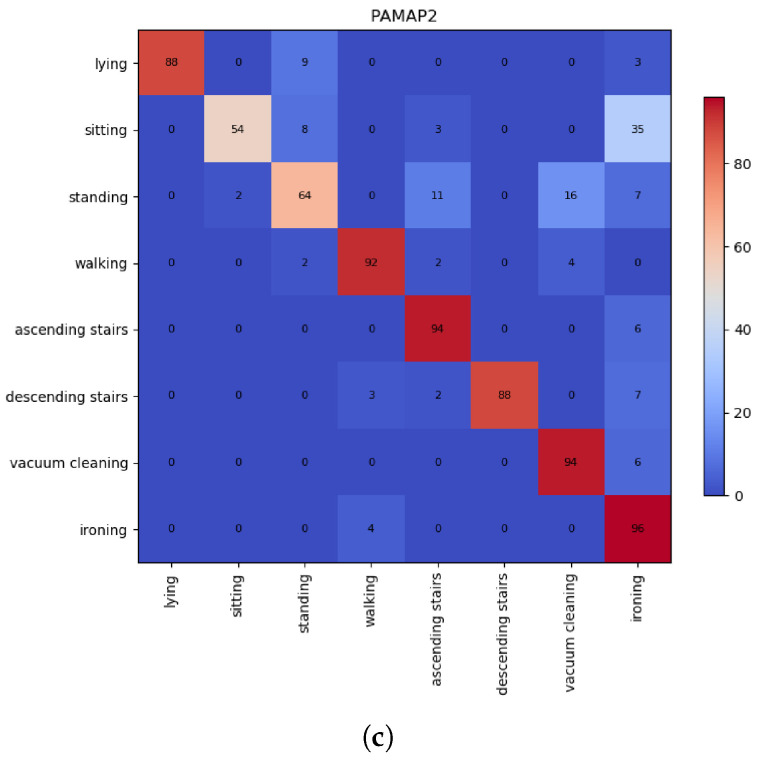
(**a**) Confusion matrix of dataset USC-HAD. (**b**) Confusion matrix of dataset DSADS. (**c**) Confusion matrix of dataset PAMAP2.

For each test domain, we conducted three sets of experiments and calculated the statistical metrics, which are presented in Table 2. The normalized confusion matrixes are illustrated in Figure 4. The experimental results demonstrate that CLEAR achieves average accuracy rates of 82.75%, 90.45%, and 81.09% on the PAMAP2, USC-HAD and DSADS datasets, respectively. In these datasets, CLEAR achieved precision rates of 85.50%, 84.48%, and 91.57%, respectively, indicating high accuracy in predicting positive instances. Correspondingly, the recall rates for CLEAR on these datasets were 82.25%, 79.88%, and 91.13%, indicating the successful identification of the majority of true positive samples. Additionally, the F-measure values for CLEAR were 82.25%, 79.88%, and 91.13% across the three datasets, indicating a good balance between precision and recall, demonstrating the model’s ability to perform well while maintaining both accuracy and recall. Based on the results, it is evident that under the multi-angle data augmentation strategy, the model’s generalization ability towards the unknown dataset has improved. Additionally, the feature fusion and contrastive learning components enable the model to capture more effective and discriminative features. Eventually, this results in high accuracy on the test set.

### 4.3. Robustness to Training Data Volume

**Figure 5:**
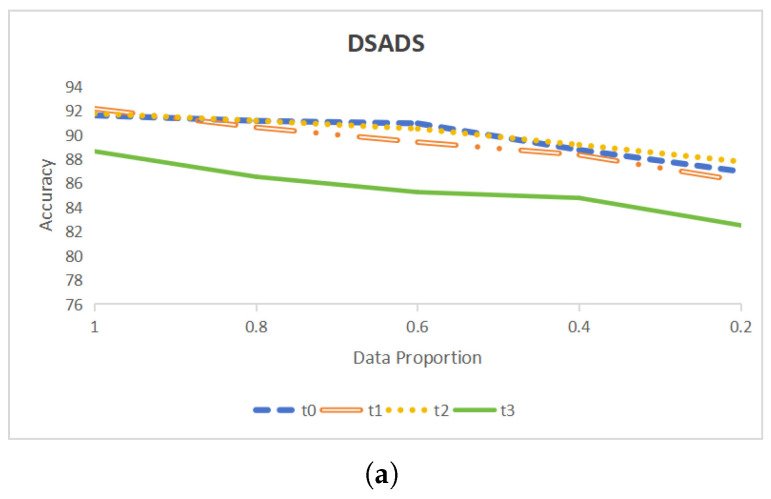

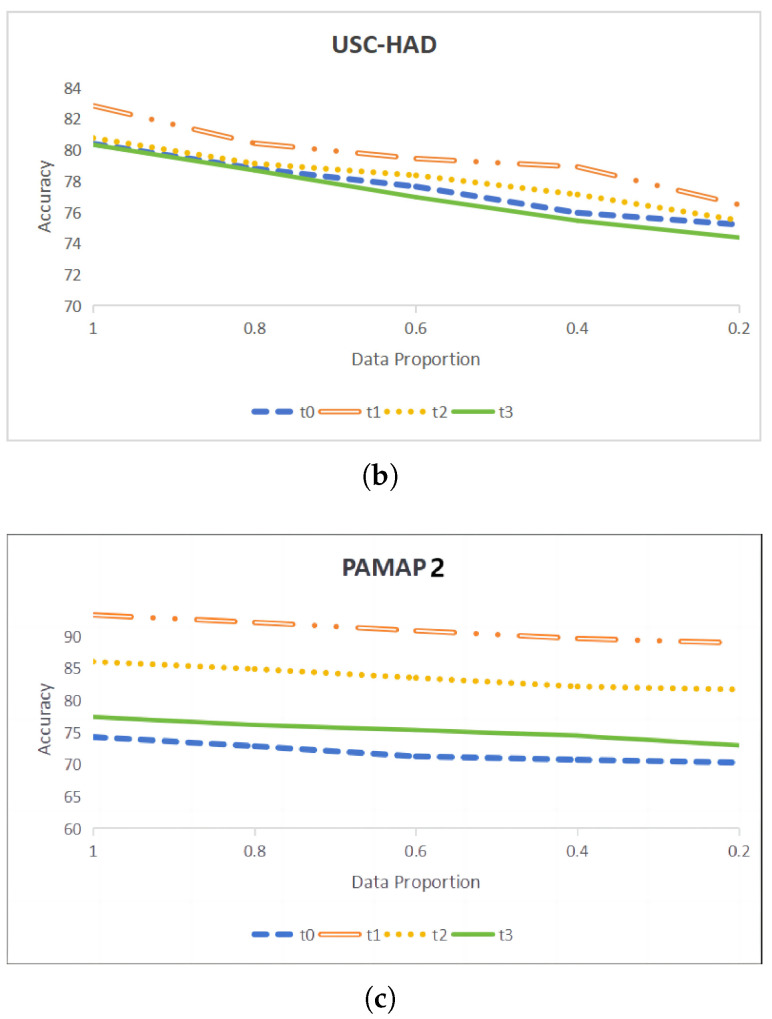
(**a**) A line graph showing the change in classification accuracy of DSADS dataset when the training data volume changes. (**b**) A line graph showing the change in classification accuracy of USC-HAD dataset when the training data volume changes. (**c**) A line graph showing the change in classification accuracy of PAMAP2 dataset when the training data volume changes.

To validate the effectiveness of CLEAR under conditions of limited labeled data, we trained the model using preprocessed data by randomly selecting proportions of 20%, 40%, 60%, 80%, and 100%, respectively. This setting approach is common and logical for several reasons. First, these proportions enable a comprehensive evaluation of the model’s performance across various data volumes. Second, 20%, 40%, 60%, and 80% are rounded percentages and equally spaced, making the results easier to interpret and accept. The experimental results are shown in Figure 5. The graph illustrates that while the training data decrease by 20%, the accuracy of the trained model on the test set decreases slowly. Specifically, for certain datasets and target sets, a decrease of 20% in training data only reduces the model’s accuracy by less than 1%. Even in the worst-case scenario, the decrease is only about 2%.

Overall, when the proportion of training data decreases from 100% to 20%, the performance of CLEAR on the DSADS, USC-HAD, and PAMAP2 datasets decreases by 5.17%, 5.70%, and 4.27%, respectively. These results indicate that CLEAR can maintain high accuracy when facing a gradual decrease in labeled training data. This also proves the robustness of CLEAR in dealing with changes in training data volume.

### 4.4. Visualization Experiment

**Figure 6:**
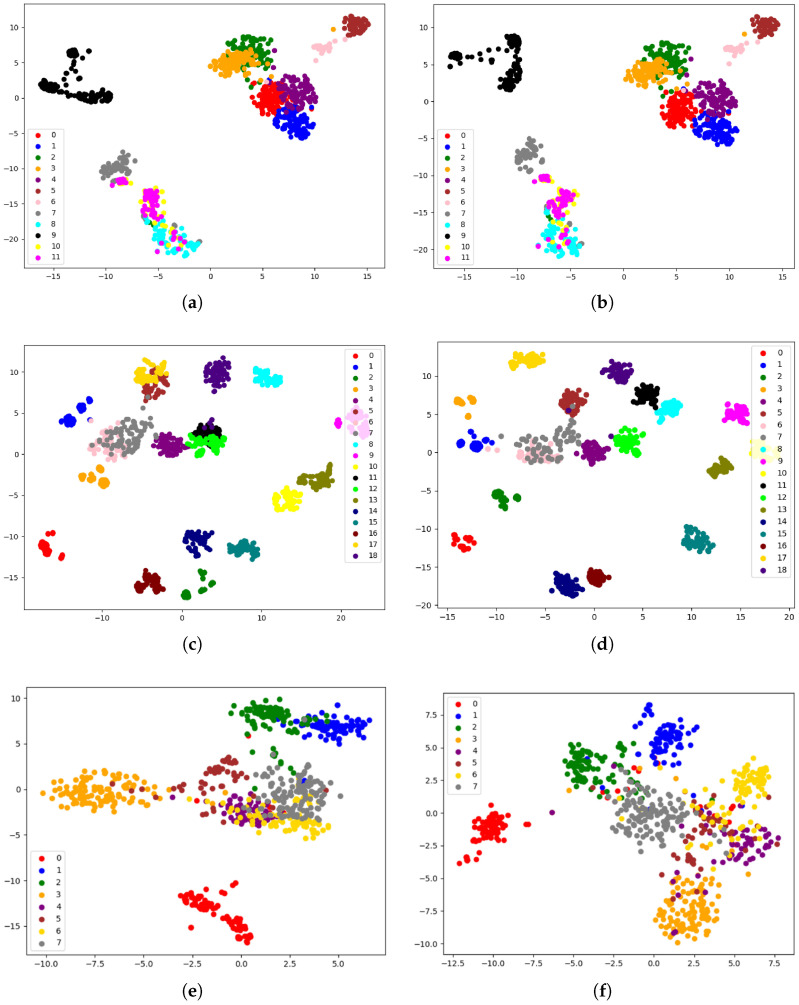
(**a**) This plot depicts the t-SNE visualization of DDLearn on the USC-HAD dataset. (**b**) This plot depicts the t-SNE visualization of CLEAR on the USC-HAD dataset. (**c**) This plot depicts the t-SNE visualization of DDLearn on the DSADS dataset. (**d**) This plot depicts the t-SNE visualization of CLEAR on the DSADS dataset. (**e**) This plot depicts the t-SNE visualization of DDLearn on the PAMAP2 dataset. (**f**) This plot depicts the t-SNE visualization of CLEAR on the PAMAP2 dataset.

To better demonstrate the advantages of our method over the baseline model, we provide visual results. Here, we particularly focus on comparing the optimal baseline DDLearn and proposed CLEAR. Different colors are used in the graph to represent samples from different classes, facilitating a more intuitive observation of their distribution in the feature space. As depicted in Figure 6, although some activities exhibit a high degree of feature distinctiveness (i.e., they are far apart from other classes in the feature space), these activities mainly comprise those that are distinctly different. However, one of the major challenges of HAR is the recognition of inter-connected and overlapped activities, such as upstairs and downstairs. As is depicted in Figure 6a,b, it can be observed that CLEAR can effectively increase the distances between activity classes when dealing with activities that are interconnected or overlap in the feature space.

The observation of the t-SNE [57] plot of the USC-HAD, particularly focusing on the red, purple, and blue clusters because of their small spacing, makes variations easier to observe. It indicates CLEAR can achieve a broader feature distribution in these regions compared to the DDLearn [51] model. This demonstrates the effectiveness of the model’s data augmentation component in overcoming the challenge of limited labeled training data. Furthermore, there is a noticeable increase in the separation between these three activity classes, resulting in minimal overlap between them. This suggests that despite data augmentation, the model’s multimodal feature extraction and contrastive learning components continue to extract more discriminative features. Consequently, this establishes a robust foundation for accurately distinguishing between various activities.

### 4.5. Ablation Study

The visualization experiment introduced in the previous section, through graphical representations of the feature space, highlighted the comparative performance of our proposed CLEAR model against the baseline DDLearn model, demonstrating how CLEAR enhances the effectiveness and distinctiveness of features. Based on these observations, we conducted an ablation study to further dissect and validate the contributions of each component within CLEAR. This systematic evaluation emphasizes the cumulative impact of the model’s components, providing a deeper understanding of how CLEAR outperforms the baseline and smoothly transitions into a detailed component analysis.

Specifically, we systematically modified the baseline model DDLearn by integrating various components, creating diverse model variants for comparison. This approach not only facilitated the evaluation of each component’s impact on model performance but also provided deeper insights into the interactions and influences among these components. Initially, we augmented the baseline model with a multimodal feature fusion component, labeled Ori + MF. Subsequently, we introduced a frequency domain enhancement module, labeled Ori + MF + FA, building upon this configuration. Finally, we incorporated a contrastive learning component, named CLEAR. Through the incremental addition of these components, we systematically explored pathways to enhance model performance, ultimately developing a comprehensive and effective methodology. This methodology enabled not only the assessment of each component’s contribution to the overall performance but also a deeper understanding of the interactions among these components.

**Figure 7:**
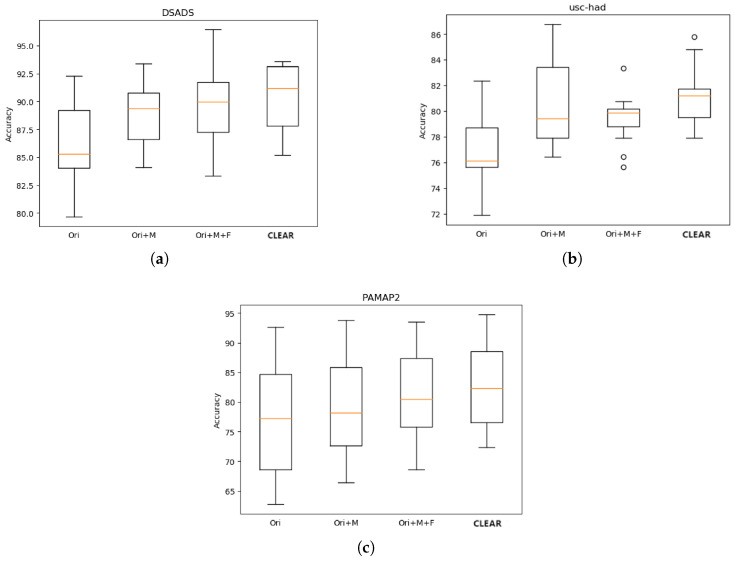
(**a**) It represents the effectiveness of CLEAR components on the DSADS dataset. (**b**) It represents the effectiveness of CLEAR components on the USC-HAD dataset. (**c**) It represents the effectiveness of CLEAR components on the PAMAP2 dataset. The components are as follows: frequency domain enhancement, feature fusion, and contrastive learning.

The boxplots presented in Figure 7 depict the results of the ablation experience conducted on three datasets. A boxplot typically consists of a box and two whiskers. The box represents the interquartile range (IQR) of the data, with the middle line indicating the median. The upper and lower edges of the box correspond to the third quartile (Q3) and the first quartile (Q1), respectively. The whiskers extend to the maximum and minimum values, with any outliers typically depicted as points outside the chart. We conducted experiments by training and testing models across three distinct target domains. Given the diversity among these domains, variations in experimental results across different domains are predictable. For instance, in the PAMAP2, the diversity of target domains leads to significant differences in experimental outcomes, resulting in excessively large box sizes. Therefore, our primary focus is on comparing the median accuracy across multiple target domains, supplemented by the edges of the box, to facilitate a comprehensive comparison across domains. Such comparative methodologies aid in gaining a more comprehensive understanding of the model performance across different datasets while accounting for the disparities between them.

Figure 7 clearly depicts the trend of gradual increase in the median values across the three datasets as we progressively introduce the proposed components. This trend indicates the effectiveness of each component in improving the model accuracy. Specifically, when integrating the multimodal feature fusion component into these three datasets, the median line shows a more substantial increase compared to other components. It is noteworthy that not only does the median line increase but also the Q1 and Q3 show significant improvements, suggesting that this component performs well across multiple training domains and experiments. The less noticeable increase in the PAMAP2 is attributed to its larger scale. This further underscores the importance of considering both the high-dimensional and multimodal nature when dealing with sensor data to effectively extract more representative features.

However, with the introduction of frequency domain enhancement, there is only a relatively small increase observed in the median line, Q1 and Q3 of the boxplots across all datasets. In the DSADS, not only does the box widen, indicating decreased stability, but also the lower bound of the boxplot is lower than before. Additionally, in the USC-HAD, although the box shrinks, indicating high model stability, its Q3 is lower than before, and even outliers emerge. It indicates that, under the unchanged model architecture, while frequency domain enhancement further diversifies the distribution of training data, it inevitably amplifies the challenge of extracting effective features. Consequently, the distinctiveness of the extracted features decreases.

We employed a contrastive learning module to address this issue, aiming to reduce intra-class distance and increase inter-class margin to improve feature distinguishability. As depicted in Figure 7, the median lines of the boxplots across all three datasets showed significant improvement. In the DSADS, while the upper quartile of the boxplot decreased compared to before, both the lower whisker and Q1 exhibited noticeable improvement. This suggests improved model performance in various scenarios, with higher average accuracy and stability despite the decrease in the upper limit. In the USC-HAD, the box became smaller, further affirming the model’s stability. Although a few outliers are present, they lie above the upper quartile, thus not adversely impacting the model’s average accuracy.

In summary, CLEAR significantly improves the performance of the baseline model, validating the effectiveness of various components in extracting meaningful and discriminative features. These results not only demonstrate the efficacy of our approach but also provide crucial insights and references for future research endeavors.

### 4.6. Comparison Study

**Figure 8:**
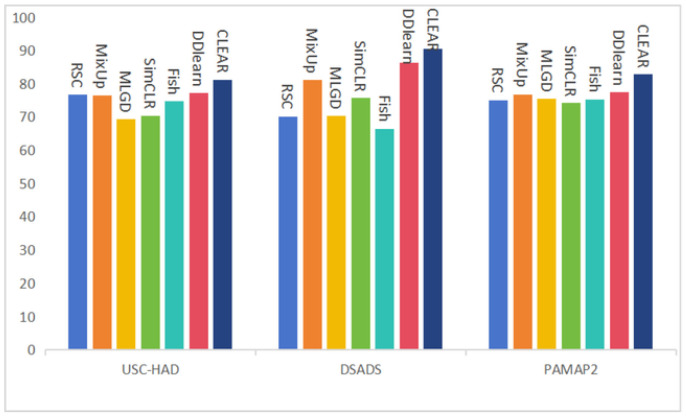
Bar chart of classification accuracy on three public Datasets in %.

We compare CLEAR with some DG/SSL methods. As shown in Table 3, the best-performing baseline model, DDLearn, attained accuracy rates of 77.15%, 86.26%, and 77.44%, respectively. This signifies that our method outperforms the best baseline models by 3.94%, 4.19%, and 5.31% on the three datasets, respectively. Additionally, it is evident from the table that our model outperforms the SimCLR baseline model by over 10% in terms of average accuracy across the three datasets. This indicates that our feature extraction method and the selection of positive and negative samples play crucial roles in enhancing the model’s performance. Furthermore, compared to the Mixup baseline model, our model achieves an average accuracy improvement of approximately 6% across the three datasets. This suggests that our data augmentation method is better suited for time series data and significantly impacts the enhancement of model performance. Moreover, to provide a more intuitive representation of the experimental results, we present bar charts, as depicted in Figure 8. These findings underscore the successful extraction of effective and discriminative features from relatively sparse labeled data, thereby enhancing the model’s generalization capability to unknown datasets. They validate the practicality and feasibility of our approach in addressing real-world problems.

## 5. Conclusions

In this paper, we present a multi-modal HAR solution via contrastive learning named CLEAR. Initially, we employ data augmentation techniques in both the temporal and frequency domains to enrich the training data distribution. Subsequently, we employ multimodal feature extraction methods to obtain more valuable feature representations. Finally, we leverage contrastive learning methods to enhance feature discriminability. CLEAR effectively mitigates issues related to poor model generalization caused by data insufficiency, suboptimal feature extraction, and low feature discriminability by utilizing data augmentation, multimodal fusion, and contrastive learning methods. We conduct extensive experiments on three public datasets to demonstrate the performance of CLEAR. The experimental results exhibit promising performance across all three datasets, validating the model’s high efficacy. Furthermore, we compare CLEAR with several out-of-domain/self-supervised learning methods, with the comparative results indicating its superiority over alternative models.

Although this study has achieved certain results, it still faces some limitations. Firstly, current data augmentation methods still generate new data based on existing datasets, which cannot fully simulate the distribution shifts in real-world scenarios. Secondly, the introduction of frequency domain data enhancement and contrastive learning modules significantly increases the time cost of the model. To address these issues, we plan to explore the integration of Generative Adversarial Network (GAN) technologies with data augmentation methods in future work. This integration aims to further expand the data distribution to better cope with the limitations of existing data. Additionally, we also plan to design a more lightweight network architecture to reduce the time cost as much as possible while maintaining model accuracy, making the model more suitable for real-time application scenarios.

## Figures and Tables

**Table 1:** Statistical information summary of three public activity recognition datasets.

Dataset	Subjects	Sensors	Positions	Data Size	Sampling Rate (Hz)
USC-HAD	14 7 F, 7 M	single Motion Node Acc, Gyro	Front right hip	2.81 M	100
DSADS	8 4 F, 4 M	Five Xsens MTx units acc, gyro, mag	torso, arms, legs	1.14 M	25
PAMAP2	9 1 F, 8 M	IMUs/HRM acc, gyro	arm, chest, ankle, mag	2.84 M	100/9

**Table 2:** Accuracy, Precision, Recall and F-measure of CLEAR on three public datasets in %.

Dataset	Accuracy	Precision	Recall	F-Measure
PAMAP2	82.76	85.50	82.87	82.25
USC-HAD	81.09	84.48	76.74	79.88
DSADS	90.45	91.57	90.84	91.13

**Table 3:** Classification accuracy on three public datasets in %.

Dataset	Mixup [39]	MLDG [58]	SimCLR [40]	Fish [59]	DDLearn [51]	CLEAR
USC-HAD	76.34	69.21	70.38	74.64	77.15	81.09
DSADS	81.07	70.30	75.65	66.49	86.26	90.45
PAMAP2	76.80	75.44	74.17	75.31	77.44	82.76

## Data Availability

The DSADS public dataset in this paper is available at https://archive.ics.uci.edu/datasets (accessed on 14 November 2024). The PAMAP public dataset in this paper is available at https://archive.ics.uci.edu/datasets (accessed on 14 November 2024). The USC-HAD public dataset in this paper is available at http://sipi.usc.edu/had/ (accessed on 14 November 2024).
